# Plant Growth and Photosynthetic Characteristics of *Mesembryanthemum crystallinum* Grown Aeroponically under Different Blue- and Red-LEDs

**DOI:** 10.3389/fpls.2017.00361

**Published:** 2017-03-17

**Authors:** Jie He, Lin Qin, Emma L. C. Chong, Tsui-Wei Choong, Sing Kong Lee

**Affiliations:** National Institute of Education, Nanyang Technological UniversitySingapore, Singapore

**Keywords:** aeroponics, LED lighting, *Mesembryanthemum crystallinum*, photosynthesis, plant growth

## Abstract

*Mesembryanthemum crystallinum* is a succulent, facultative crassulacean acid metabolism (CAM) plant. Plant growth and photosynthetic characteristics were studied when *M. crystallinum* plants were grown indoor under light emitting diodes (LED)-lighting with adequate water supply. Plants were cultured aeroponically for a 16-h photoperiod at an equal photosynthetic photon flux density of 350 μmol m^-2^ s^-1^ under different red:blue LED ratios: (1) 100:0 (0B); (2) 90:10 (10B); (3) 80:20 (20B); (4) 70:30 (30B); (5) 50:50 (50B); and (6)100:0 (100B). *M. crystallinum* grown under 10B condition had the highest shoot and root biomass and shoot/root ratio while those grown under 0B condition exhibited the lowest values. Compared to plants grown under 0B condition, all other plants had similar but higher total chlorophyll (Chl) and carotenoids (Car) contents and higher Chl *a*/*b* ratios. However, there were no significant differences in Chl/Car ratio among all plants grown under different red- and blue-LEDs. Photosynthetic light use efficiency measured by photochemical quenching, non-photochemical quenching, and electron transport rate, demonstrated that plants grown under high blue-LED utilized more light energy and had more effective heat dissipation mechanism compared to plants grown under 0B or lower blue-LED. Statistically, there were no differences in photosynthetic O_2_ evolution rate, light-saturated CO_2_ assimilation rate (*A*_sat_), and light-saturated stomatal conductance (*g*_ssat_) among plants grown under different combined red- and blue-LEDs but they were significantly higher than those of 0B plants. No statistically differences in total reduced nitrogen content were found among all plants. For the total soluble protein, all plants grown under different combined red- and blue-LEDs had similar values but they were significantly higher than that of plants grown under 0B condition. However, plants grown under higher blue-LEDs had significant higher ribulose-1,5-bisphosphate carboxylase oxygenase (Rubisco) protein than those plants grown under lower blue-LED. High *A*_sat_ and *g*_ssat_ but very low CAM acidity of all *M. crystallinum* plants during light period, imply that this facultative CAM plant performed C_3_ photosynthesis when supplied with adequate water. Results of this study suggest that compared to red- or blue-LED alone, appropriate combination of red- and blue-LED lighting enhanced plant growth and photosynthetic capacities of *M. crystallinum*.

## Introduction

*Mesembryanthemum crystallinum* (common name: ice plant) is a succulent, facultative crassulacean acid metabolism (CAM) plants native to Europe and South Africa. Large bladder cells covering the leaf of *M. crystallinum* are enlarged epidermal cells that functions to reserve water and to store accumulated salt (Adams et al., 1998). When grown under unstressed conditions, stomata of C_3_ and C_4_ plants open in the light and close in darkness. However, for facultative CAM plants, their stomata shift from diurnal to nocturnal opening during stress-induced shifts from C_3_ to CAM metabolism (Osmond, 1978). *M. crystallinum* plants have been commonly studied for switching between C_3_ photosynthesis and CAM photosynthesis in response to environmental stresses including high salinity (Winter et al., 1982; Abd El-Gawad and Shehata, 2014), drought (Adams et al., 1998; Bohnert and Cushman, 2000), anoxia and exposure of roots to low temperatures (Winter and Holtum, 2007), high temperature (Kluge and Ting, 1978).

The edible leaves of the *M. crystallinum* plants contain high nutritional values and were successfully grown inside greenhouses in Japan and Taiwan under cool temperature. The growth of these halophytic plants were mostly studied in different treatments of soil (Winter and Holtum, 2007). To enhance local vegetable production, recently we have successfully grown them in land scarce Singapore using an indoor aeroponic farming system with adequate water supply under light emitting diodes (LEDs) lighting. However, there is very little work done on its photosynthetic characteristics when *M. crystallinum* is grown under LED lighting. Plant growth and photosynthesis is strongly influenced by blue or red light (Bula et al., 1991; Yorio et al., 2001; Darko et al., 2014; He et al., 2015; Hernández and Kubota, 2016; Ooi et al., 2016; Shengxin et al., 2016; Wang et al., 2016). It was observed that lettuce and other dicotyledonous plants developed excessive hypocotyl and stem elongation, leaf extension, and reduced chlorophyll (Chl) when grown under red-LED as the sole source of irradiation. The abnormal morphological characteristics were eliminated when red-LED was supplemented with blue light (Bula et al., 1991; Yorio et al., 2001; Nhut et al., 2003; Li et al., 2013; Hernández and Kubota, 2016; Shengxin et al., 2016; Wang et al., 2016). Yield of lettuce, spinach, and radish crops grown under appropriate combination of red and blue light was enhanced compared to red light alone (Yorio et al., 2001). Similar results were found in strawberry plantlet (Nhut et al., 2003), rapeseed (*Brassica napus* L.) plantlets *in vitro* (Li et al., 2013), rapeseed rosette leaves (Shengxin et al., 2016), and cucumber seedlings (Hernández and Kubota, 2016; Trouwborst et al., 2016). In a recent developed highly energy-efficient laser-illuminated growth chamber, Ooi et al. (2016) also showed that the application of diffused single-wavelength red and blue laser light is adequate for the growth and development of *Arabidopsis thaliana*.

The use of red-LED light to drive photosynthesis has been widely accepted due to (i) red wavelengths (600–700 nm) are efficiently absorbed by photosynthetic pigments and (ii) early LEDs were red close to an absorption peak of Chl (Sager and McFarlane, 1997). However, plants grown under blue light have higher Chl *a*/*b* ratio, greater cytochrome (Cyt) *f* and ribulose-1,5-bisphosphate carboxylase oxygenase (Rubisco) contents than plants grown under red light (López-Juez and Hughes, 1995). Compared to lettuce plants grown under green- and red-LED, high intensity of blue-LED enhanced Rubisco content (Muneer et al., 2014). Growth of cucumber under red light alone, in the absence of blue light, led to dysfunction of the photosynthetic machinery, in particular a loss of photochemical efficiency of photosystem II (PSII), the maximum photosynthetic capacity per leaf area, and the Chl content. Only 7% blue light was sufficient to prevent any overt dysfunction in photosynthesis (Hogewoning et al., 2010). Recent studies reported that high intensity blue-LEDs promote plant growth by controlling stomatal movement and enhanced the expression of PSII-core dimer and PSII-core monomer (Muneer et al., 2014; Wang et al., 2016), maintaining the integrity of chloroplast proteins (Muneer et al., 2014).

Stomatal movements can be affected by various environmental factors including light. Sharkey and Raschke (1981) observed that stomatal opening was most responsive to light in the blue region of the spectrum than other wavelengths. Zeiger and Hepler (1977) reported that blue light was nearly 10 times more effective than red light to induce opening of stomata. Using the obligate CAM bromeliad, *Aechmea* “Maya,” a recent study has shown that of the different light treatments (blue, green, and red light), imposed low-fluence blue light was a key determinant in regulating stomatal responses (Ceusters et al., 2014). It was previously reported that stomata isolated from *M. crystallinum* operating in the C_3_ mode responded to blue but not to red light (Mawson and Zaugg, 1994). Tallman et al. (1997) have also shown that the stomatal response of *M. crystallinum* in the C_3_ mode depended solely on the guard cell response to blue light. It has been hypothesized that the xanthophyll, zeaxanthin play an important role in blue light photoreception of guard cells. Changes in the regulation of the xanthophyll cycle in guard cells parallel the shift from diurnal to nocturnal stomatal opening associated with CAM induction (Tallman et al., 1997).

The stimulation of stomatal opening by blue light may contribute to the enhancement of productivity (Sharkey and Raschke, 1981; Karlsson, 1986; Hogewoning et al., 2010; Darko et al., 2014; Muneer et al., 2014). Goins et al. (1997) also reported that in wheat, photosynthetic rates were higher in leaves under red-LED combined with blue light. They suggested that the increase in photosynthetic rates may resulted from the increased stomatal conductance (*g*_s_). However, Yorio et al. (2001) reported that photosynthetic rates were not increased when stomotal opening was stimulated under red-LED supplemented with blue light. In their study with lettuce plants, Kim et al. (2004) concluded that *g*_s_ was responsive to spectral quality during growth and, in the short-term, was not directly coupled to dry matter accumulation. Wang et al. (2016) found that photosynthetic rate of lettuce leaves increased with increasing blue to red-LED ratio until 1, associated with increased *g*_s_ and stomatal density. However, shoot dry weight (DW) increased with decreasing blue- to red-LED ratio. The increase of shoot DW mainly resulted from increasing leaf number and leaf area. Similar treads were also found in rice (Matsuda et al., 2004), cucumber (Hogewoning et al., 2010; Hernández and Kubota, 2016) and rapeseed rosette leaves (Shengxin et al., 2016). Therefore, the effect of blue light and the blue- to red-LED ratio on leaf photosynthesis and dry matter productivity remain unclear and the effects may be species-dependent.

The aim of the present work was to investigate the growth, photosynthetic pigments, photosynthetic light utilization and gas exchange and CAM acidity, total reduced nitrogen (TRN), soluble protein and Rubisco protein of *M. crystallinum* grown under different combinations of blue- and red-LEDs using an indoor aeroponic farming system. The findings of this project help advance existing understanding of *M. crystallinum* plant physiology under soilless culture and artificial lightings. It also provides the growers with basic scientific information to enhance productivity of *M. crystallinum* at low production cost through the cost effective LED lighting.

## Materials and Methods

### Plant Materials and Culture Methods

**FIGURE 1:**
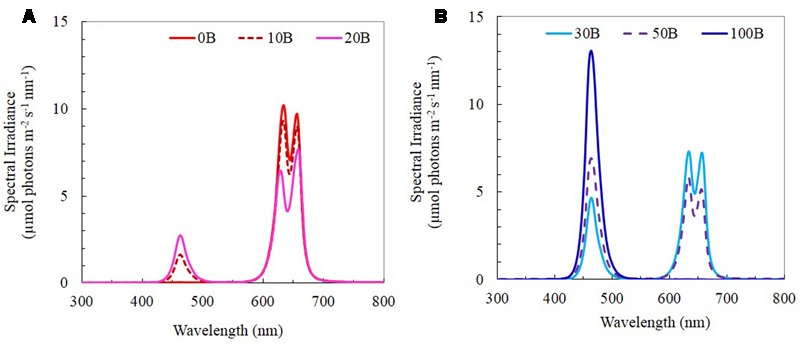
**(A)** Light spectral of 0B, 10B, and 20B lighting conditions. **(B)** Light spectral of 30B, 50B, and 100B lighting conditions. Spectral scans were recorded every 0.5 nm with a spectroradiometer (PS300, Apogee Instruments, USA).

Seeds of *M. crystallinum* were germinated on filter papers and inserted into polyurethane cubes for a month of incubation under dim natural sunlight for 4 weeks. The seedlings were subsequently transplanted onto the indoor vertical aeroponic farming systems. The plants were then cultured with a 16-h photoperiod of light and exposed to an equal photosynthetic photon flux density (PPFD) of 350 μmol m^-2^ s^-1^ under each of the six red:blue LED ratios (i.e., PPFD ratios) (1) 100:0; (2) 90:10; (3) 80:20; (4) 70:30; (5) 50:50; and (6) 0:100, which were named 0B, 10B, 20B, 30B, 50B, and 100B, respectively. All LEDs were designed by our research team and they were tailor-made by the local contractor (Dissis LED Lighting Technology, Singapore) in collaboration with their business partner from China. The spectra of the six red- and blue-LEDs are shown in **Figures 1A,B**. Temperature and relative humidity in the growth room were maintained at 26°C/28°C (day/night) and 48%/52% (day/night), respectively. Plants were supplied with full strength nutrient solution of Netherlands Standard Composition and electrical conductivity and pH were maintained at 2.0 ± 0.2 mS and 6.5 ± 0.5, respectively. The samples were harvested after transplanting for 21 days.

### Measurement of FW and DW of Root and Shoot

After 21 days of transplanting, four *M. crystallinum* plants under each LED treatment were harvested. The polyurethane cube was carefully removed from the roots. The shoot and root were blotted dry and weighed separately to determine their fresh weight (FW). All samples were then wrapped individually in aluminum foil, dried at 80°C for 4 days before measuring their DW.

### Measurements of Photosynthetic Pigments

Fresh leaf sections of 0.1 g cut from fully expanded leaves were soaked in 5 ml of N,N-dimethylformamide (Sigma-Aldrich) in the dark for 48 h, at 4°C. The absorbance was read at 480, 647, and 664 nm, using a spectrophotometer (UV-2550 Shimadzu, Japan). The contents of Chl *a*, Chl *b*, and carotenoids (Car) were calculated according to Wellburn (1994).

### Measurement of qP, NPQ, and ETR

Leaf discs (1 cm diameter) were punctured from the leaves previously used for the determination of photosynthetic pigments and placed on moist filter papers in Petri dishes. They were pre-darkened for 15 min prior to measurements. Via the Imaging-PAM Chlorophyll Fluorometer (Walz, Effeltrich, Germany), images of fluorescence emission were digitized within the camera and transmitted via a Firewire interface (400 Mb/s) (Firewire-1394.com, Austin, TX, USA) to a personal computer for storage and analysis. Measurements and calculations of electron transport rate (ETR), photochemical quenching (qP), and non-photochemical quenching (NPQ) was determined according to He et al. (2011).

### Measurement of CO_2_-Saturated Photosynthetic O_2_ Evolution Rate

For facultative CAM plants, reliable determinations of light-use characteristics of leaf were preferably carried out at saturated CO_2_ concentration. The light response curves of O_2_ evolution rate were determined using a leaf disc O_2_ electrode (CBID, Hansatech, King’s Lynn, Norfolk, UK). A leaf discs was placed under saturating CO_2_ condition (1% CO_2_ from 1 M carbonate/bicarbonate buffer, pH 9) and illuminated with different PPFDs. The photosynthetic light response curves determined by O_2_ evolution were measured under 0, 10, 30, 40, 60, 90, 150, 200, 250, 400, 600, 820, 1,000, 1,200, 1,500, and 1,900 μmol photons m^-2^ s^-1^.

### Titratable Acidity Determination

The CAM acidity of leaves was determined immediately before and after the 16 h photoperiod, respectively according to He and Teo (2007). Five leaf discs of 7 mm in diameter were punched out of each leaf sample with a core borer and they were then transferred into test tubes containing 1 ml of distilled water (neutral pH). The tubes were then immersed into a boiling water-bath for 15 min and then allowed to cool to room temperature. The extract was subsequently titrated against 0.01 M sodium hydroxide solution, NaOH(aq), using three drops of phenolphthalein as an indicator until the end-point (pale pink coloration) was reached. The volume of NaOH(aq) required to reach the end-point of titration was recorded. The plant material was then wrapped in an aluminum foil and dried in an oven at 80°C for 1 week before the DW is measured. Titratable acidity was calculated using the formula: [0.01× volume of NaOH(aq)]/DW. The fluctuation of CAM acidity was calculated by obtaining the difference between titratable acidity immediately before and after the 16 h photoperiod.

### Measurements of *A*_sat_ and *g*_ssat_

The light-saturated CO_2_ assimilation rate (*A*_sat_) and light-saturated *g*_s_ (*g*_ssat_) of fully expanded leaves were measured simultaneously in the growth room after plants were exposed to different LEDs for 3–4 h, with an open infrared gas analysis system with a 6 cm^2^ chamber (LI-6400, Biosciences, USA). Readings were taken with a LED light source, which supplied 1000 μmol m^-2^ s^-1^ of PPFD. Light response curves of CO_2_ fixation from intact leaves under ambient CO_2_ showed that a PPFD of 1000 μmol m^-2^ s^-1^ was saturating for *M. crystallinum* photosynthesis (data not shown). The light source emitted in the wavelength ranged between 420–510 and 610–730 nm. The spectral output of the light source has one peak centered at about 465 nm and second peak centered at about 670 nm. Average ambient [CO_2_] and relative humidity in the chamber were 415 ± 5 μmol mol^-1^ and 70%, respectively. Measurements were recorded when both *A*_sat_ and *g*_ssat_ were stable.

### Measurement of TRN

It was determined by Kjeldahl digestion using 0.05 g of dried leaf tissue samples in 5 ml of concentrated sulfuric acid with a tablet of Kjeltabs at 350°C until the mixture turned clear (Allen, 1989). After the digestion was completed, the mixture was allowed to cool for 30 min before it was used to determine N content by a Kjeltec auto 2300 analyzer. The content of TRN present in the sample was calculated as a unit of mg g^-1^ DW.

### Measurement of Leaf TSP and Rubisco Protein Contents

Fresh leaf sample of 1 g was ground in liquid nitrogen. One milliliter of extraction buffer [100 mM Bicine-KOH (pH 8.1), 20 mM MgCl_2_, 2% polyvinylpyrrolidone (PVP) buffer (Lowry et al., 1951)] was added to the mixture to grind. Another 2 ml was added before pouring the grounded mixture into a centrifuge tube. The mixture was centrifuged for 30 min at 4°C using a 70 Ti rotor at 35,000 rpm (about 10,000 *g*) (Beckman ultracentrifuge Optima XL-100K). A total of 500 μl aliquot of the supernatant was pipetted and mixed thoroughly with 500 μl of solubilizing solution (20% glycerol, 0.02% bromophenol blue, 5% sodium dodecyl sulfate (SDS), 0.125 M Tris and 10% β-mercaptoethanol) before storing at -20°C for quantifying Rubisco protein. Another 1 ml aliquot of the supernatant pipetted was mixed with 4 ml of 80% cold acetone before centrifuging for 10 min at 4000 rpm. The amount of total soluble protein (TSP) present was determined using the method according to Lowry et al. (1951). Rubisco protein present in the samples was quantified using sodium dodecyl sulfate polyacrylamide gel electrophoresis (SDS-PAGE). Solubilized protein was boiled for 5 min and loaded onto the Mini-PROTEAN Precast Gel (TGX gel, any kD, BIO-RAD, USA). A 192 mM glycine, 3.47 mM SDS, 25 mM Tris–HCl (pH 8.6) running buffer was used in running of gels for 30 min. The separated proteins were stained for 3 h in a mixture (0.2% Coomassie Brilliant Blue in 10% acetic acid, 50% methanol) and destained in a destainer (7% acetic acid and 25% ethanol). The separated proteins stained were analyzed using a FluorChem 8800 gel imagine system under visible light. Areas of large subunit and small subunit were calculated in terms of Rubisco content according to a standard (He et al., 2013).

### Statistical Analysis

One-way analysis of variance (ANOVA) was used to test for significant differences of different variances crossed with the six different light treatments. Tukey’s multiple comparison tests were used to discriminate the means (MINITAB, Inc., February 17, 2013).

## Results

### FW and DW of Shoot and Root

**FIGURE 2:**
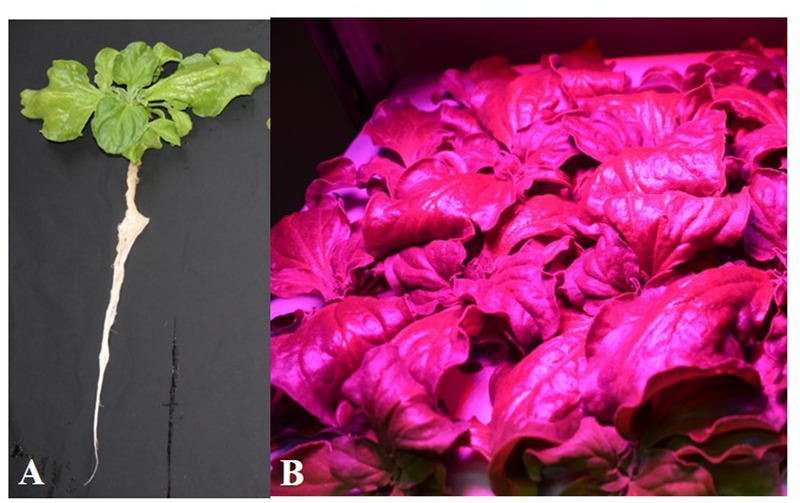
***Mesembryanthemum crystallinum* grown under 10B conditions for 21 days. (A)** A plant after removing from the growing system; **(B)** plants grown on an indoor aeroponic farming system under 10B condition.

**FIGURE 3:**
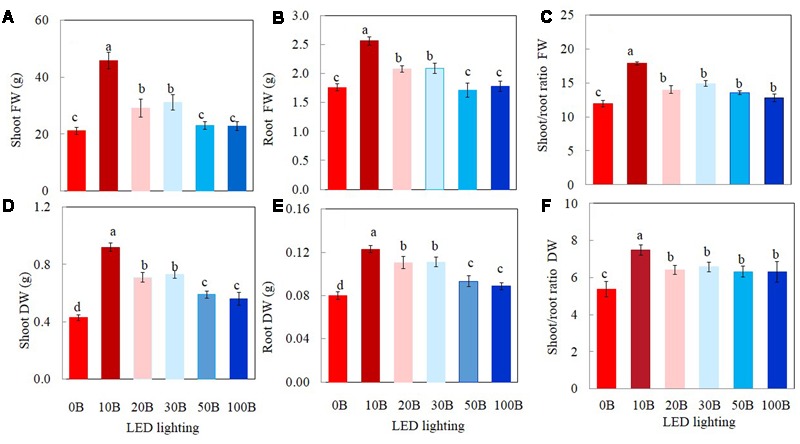
**Shoot FW and DW (A,D)**, root FW and DW **(B,E)**, and shoot/root ratio FW and DW **(C,F)** of *M. crystallinum* grown under different combinations of red- and blue-LED lightings for 21 days. Values are means ± standard errors. Means with different letters are statistically different (*P* < 0.05; *n* = 6) as determined by Tukey’s multiple comparison test.

All plants grew well and appeared healthy when they were growing indoor using vertical aeroponic farming systems under different combinations of red- and blue-LED lightings. **Figure 2** shows a 21-day *M. crystallinum* plant (**Figure 2A**) and those plants (**Figure 2B**) were still grown on an aeroponics system under 10B conditions just before harvest. At harvest, the shoot, root FW and DW of *M. crystallinum* plants were higher under 10B, 20B, and 30B conditions than under 0B, 50B, and 100B conditions (**Figures 3A,B,D,E**). Plants grown under the 10B treatment had the highest values of shoot, root FW and DW while there were no significant differences between 20B and 30B treatments. Plants grown under 0B, 50B, and 100B conditions had similar lower values of shoot, root FW and DW. Compared to plants grown under 0B condition, all other plants grown under different combinations of red- and blue-LED generally had higher shoot/root ratio FW and DW with the highest value obtained from 10B treatment (**Figures 3C,F**).

### Photosynthetic Pigments

**FIGURE 4:**
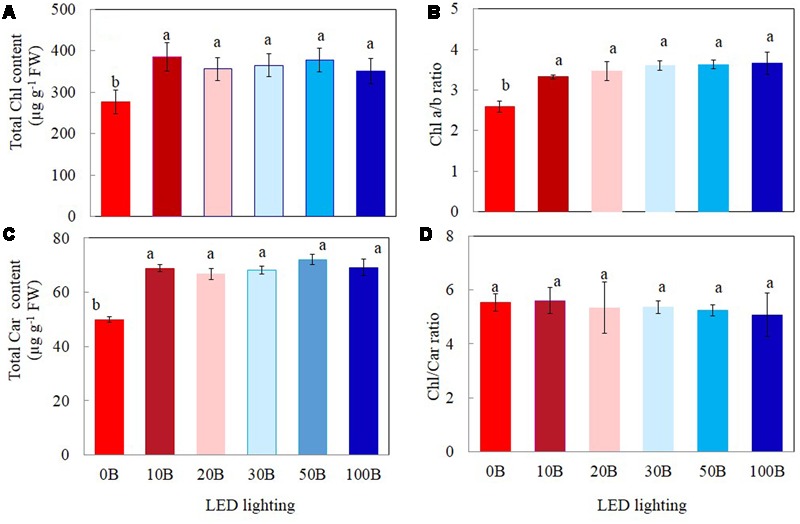
**Total Chl content (A)**, Chl *a*/*b* ratio **(B)**, total Car content **(C)**, and Chl/Car ratio **(D)** of *M. crystallinum* grown under different combinations of red- and blue-LED lightings for 21 days. Values are means ± standard errors. Means with different letters are statistically different (*P* < 0.05; *n* = 4) as determined by Tukey’s multiple comparison test.

Plants grown under 0B had lower total Chl (**Figure 4A**), total Car contents (**Figure 4C**), and Chl *a*/*b* ratio (**Figure 4B**) by 27, 28, and 19%, respectively, compared to other plants grown under different combination of red- and blue-LED lightings. There were no significant differences in Chl/Car ratios for all plants (**Figure 4D**).

### Photochemical Light Use Efficiency Measured by qP, NPQ, and ETR

**FIGURE 5:**
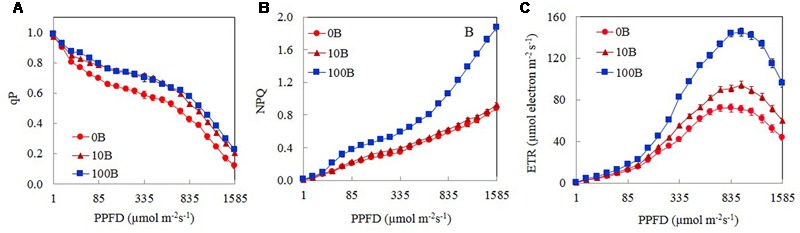
**Light response curves of qP (A)**, NPQ **(B)**, and ETR **(C)** of *M. crystallinum* grown under 0B, 10B, and 100B conditions for 21 days. Each point is the mean of 20 measurements of four different leaves from four different plants (*n* = 20). Values are means ± standard errors.

**Figure 5** shows the light response curves of qP, NPQ, and ETR from *M. crystallinum* plants grown under 0B, 10B, and 100B. For all plants, qP decreased with increasing PPFD from 15 to 1585 μmol m^-2^ s^-1^ (**Figure 5A**). There were no significant differences in qP values between 10B and 100B plants regardless of measuring PPFD. From a low PPFD of 40 μmol m^-2^ s^-1^ onward, qP of *M. crystallinum* plants grown under 0B condition was significantly lower than those of plants grown under 10B and 100B conditions under the same PPFD (**Figure 5A**). The NPQ values for all plants increased gradually with increasing PPFDs from 15 to 1585 μmol m^-2^ s^-1^ (**Figure 5B**). *M. crystallinum* plants grown under 100B conditions had much higher NPQ values compared to those grown under 0B and 10B conditions at a PPFD of 40 μmol m^-2^ s^-1^ onward. Measured at the highest PPFD of 1585 μmol m^-2^ s^-1^, the NPQ value of *M. crystallinum* plants under 100B was almost twofold higher than those of plants under 0B and 10B conditions (**Figure 5B**). ETR increased with increasing PPFD from 15 to 1105 μmol m^-2^ s^-1^ (**Figure 5C**) and decreased with further increasing PPFD beyond 1150 μmol m^-2^ s^-1^. Although the light response curves were similar for all plants, at a moderate PPFD of 155 μmol m^-2^ s^-1^ onward, the ETR values for plants grown under 100B treatments were significantly higher than plants grown under 0B and 10B treatments.

**FIGURE 6:**
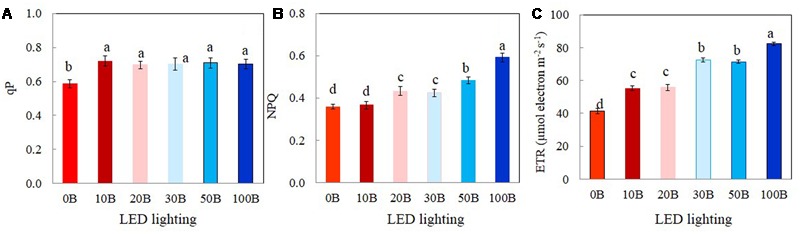
**qP (A)**, NPQ **(B)**, and ETR **(C)** measured at a PPFD of 335 μmol photon m^-2^ s^-1^ from *M. crystallinum* grown under combined red- and blue-LED lightings for 21 days. Each bar is the mean of 20 measurements of four different leaves from four different plants. Values are means ± standard errors. Means with different letters are statistically different (*P* < 0.05; *n* = 20) as determined by Tukey’s multiple comparison test.

The values of qP, NPQ, and ETR measured at a PPFD of 335 μmol m^-2^ s^-1^, which was close to their growth PPFD are shown in **Figure 6**. There were no significant differences in qP values among plants grown under 10B–100B conditions. However, qP values of *M. crystallinum* plants grown under 0B condition was significantly lower than all other plants (**Figure 6A**). *M. crystallinum* plants growth under 100B had the highest NPQ followed by those grown under 50B and 30B and then 20B and 10B conditions. The lowest NPQ was obtained from plants grown under 0B conditions (**Figure 6B**). The differences in ETR of *M. crystallinum* plants grown under different combinations of red- and blue-LED were similar (**Figure 6C**) to those of NPQ (**Figure 6B**).

### Photosynthetic Gas Exchanges, *g*_s_, and CAM Acidity

**FIGURE 7:**
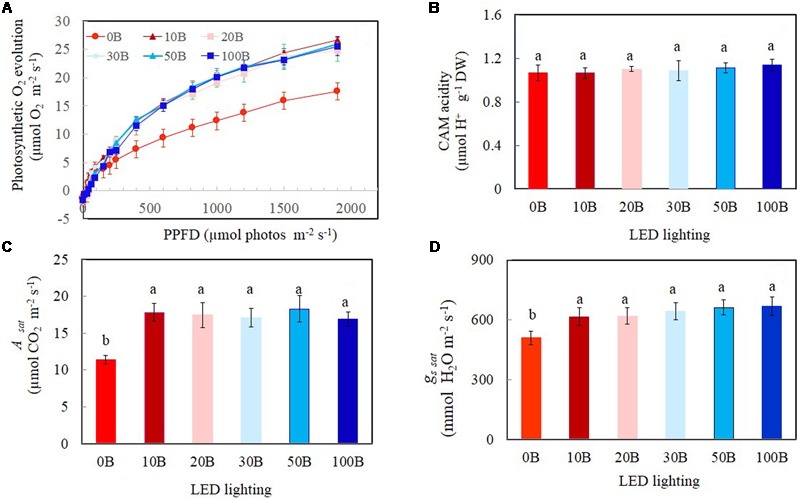
**Light response curves of photosynthetic O_2_ evolution (A)**, crassulacean acid metabolism (CAM) acidity **(B)**, *A*_sat_ **(C)**, and *g*_s__sat_ **(D)** of *M. crystallinum* grown under different combined red- and blue-LED lightings for 21 days. Values are means ± standard errors. For panels **(B–D)**, means with different letters are statistically different (*P* < 0.05; *n* = 4) as determined by Tukey’s multiple comparison test.

Light response curves of photosynthetic O_2_ evolution were determined from all plants grown under the six different red- and blue-LED lightings. Photosynthetic O_2_ evolution rates increased with increasing PPFD from 15 to 1499 μmol m^-2^ s^-1^ (**Figure 7A**). There were no significant differences in photosynthetic O_2_ evolution rates among different plants when they were measured under PPFDs below 90 μmol m^-2^ s^-1^. Thus, all plants had similar quantum yields (data not shown). However, measured from a PPFD of 250 μmol m^-2^ s^-1^ and above, plants grown under 0B had significantly lower photosynthetic O_2_ evolution rate compared to all other plants which showed similar values (**Figure 7A**). There were no significant differences for CAM acidity across all treatments (**Figure 7B**). Compared to plants grown under 0B condition, all other plants had similar values of *A*_sat_ (**Figure 7C**) and *g*_ssat_ (**Figure 7D**) but they were significantly higher than that of plants grown under 0B condition.

### TRN, Total Leaf Soluble Protein, and Rubisco Protein

**FIGURE 8:**
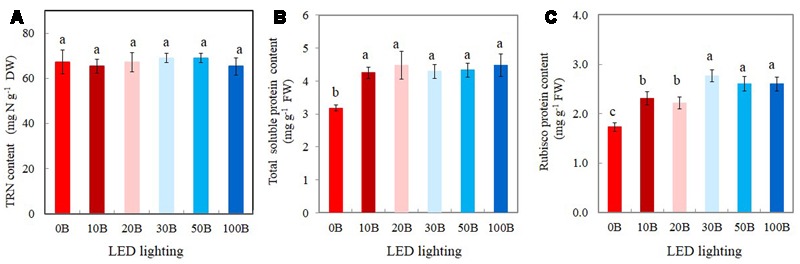
**Total reduced nitrogen, TRN (A)**, total leaf soluble protein **(B)**, and Rubisco protein contents **(C)** of *M. crystallinum* grown under different combined red- and blue-LED lightings for 21 days. Values are means ± standard errors. Means with different letters are statistically different (*P* < 0.05; *n* = 4) as determined by Tukey’s multiple comparison test.

There were no statistically differences in TRN content among all plants (**Figure 8A**). For the TSP contents, although all plants grown under different combined red- and blue-LED had similar values they were significantly higher than that of plants grown under 0B condition (**Figure 8B**). For Rubisco, the plants grown under high percentage of blue-LED such as 30B, 50B, and 100B had similarly higher content than 10B and 20B conditions. *M. crystallinum* grown under without blue-LED such as 0B condition had the lowest content of Rubisco protein (**Figure 8C**).

## Discussion

Although it is currently an intensive research area, application of red- and blue-LEDs to cultivate crops indoor seems to be the most challenging for horticultural researchers (Hogewoning et al., 2010; Darko et al., 2014; Muneer et al., 2014; He et al., 2015; Wang et al., 2016). Recently, Ooi et al. (2016) has also demonstrated the use of high-powered single-wavelength red and blue laser lights for indoor horticulture using *Arabidopsis*. However, the potential application of laser light for sustainable food production needs further research with more crops. This is because not only light intensity but also light quality and duration affect photosynthesis, morphology, and growth (Goins et al., 1997; Yorio et al., 2001; Matsuda et al., 2004; Cope and Bugbee, 2013; He et al., 2016; Shengxin et al., 2016; Wang et al., 2016). Another crucial role of light quality in regulating photosynthesis is through stomatal opening and closing. Plants grown under blue light rich environment had increased *g*_s_ that resulted in greater net photosynthetic rate than those grown under blue light limited conditions (Matsuda et al., 2004; Hogewoning et al., 2010; Shengxin et al., 2016; Wang et al., 2016). However, due to its strong influence on leaf morphology that resulted in reducing light interception, increase in *g*_s_ does not always enhance growth (Kim et al., 2004; Hogewoning et al., 2010). *M. crystallinum*, a facultative CAM plant has been successfully grown in our indoor farming systems aeroponically with adequate water supply under LED lighting. However, very little is known about the effects of light quality on its growth and photosynthetic characteristics.

In the present study, results of shoot, root FW and DW showed that 10B was the best combination of red- and blue-LED followed by 20B and 30B (**Figures 3A–D**) compared to those plants grown under 0B (red-LED alone). These results correspond to most studies that the combination of red and blue light is important for leaf expansion and biomass accumulation (Hogewoning et al., 2010; Johkan et al., 2010; Shengxin et al., 2016; Wang et al., 2016). For instance, a combination of red- and blue-LED enhanced the fresh and dry matter productivity of plant species including rice (Matsuda et al., 2004), wheat (Goins et al., 1997), spinach, radish, and lettuce (Yorio et al., 2001; Wang et al., 2016); strawberry (Nhut et al., 2003); rapeseed (Li et al., 2013; Shengxin et al., 2016); and cucumber (Hogewoning et al., 2010; Hernández and Kubota, 2016). However, in the present study, there were no significant differences in shoot and root productivities between the conditions of stronger blue-LEDs such as 50B and 100B and 0B (100% red-LED) conditions (**Figures 3A–D**). In this study, it was also shown that combinations of red- and blue-LED generally increased shoot/root ratio FW and DW with the highest value obtained from plants grown under 10B conditions (**Figures 3C,F**), indicating that increase in shoot biomass was greater than in root biomass. However, it was reported that the shoot/root ratio of lettuce plants irradiated with blue-containing LED lights compared to red-LED or fluorescence light decreased due to the greater increase in root biomass (Johkan et al., 2010). In the study of blue light dose-response curves of cucumber leaves, Hogewoning et al. (2010) reported that the leaf mass per unit leaf area (LMA) increased with blue percentage from 0 to 50%. In this study, all combined red- and blue-LEDs enhanced LMA of *M. crystallinum* compared to those plants grown under red-LED alone. However, there was no clear trend in LMA among *M. crystallinum* grown under different combinations of red- and blue-LED (data not shown). In the study with lettuce plants, blue-LEDs in combination with high light intensity was important for growth elongation and biomass accumulation compared to plants grown under low light intensities (Muneer et al., 2014). In another study with lettuce, Wang et al. (2016) studied leaf morphology and shoot DW with different red/blue light ratios of 12, 8, 4, and 1. They reported that shoot DW increased with increasing red/blue light ratio, which was mainly due to increased leaf number and leaf area under the higher red/blue light ratio. It was also reported that the optimal red/blue light ratios for FW and DW accumulation were 7/3 in strawberry plantlet (Nhut et al., 2003) and 1/3 in rapeseed plantlets *in vitro* (Li et al., 2013), respectively, but 9 in cucumber seedlings (Hernández and Kubota, 2016), spinach (Yorio et al., 2001), lettuce (Bula et al., 1991; Yorio et al., 2001; Stutte et al., 2009), and *Arabidopsis* (Ooi et al., 2016). Based on the above discussion, blue light promote growth by stimulating morphological response. However, the optimal blue light fraction may depend on species or other experimental conditions.

The different optimal red to blue light ratios for different species could also be due to the experiments were carried out at the different growth stages such as plantlets of strawberry and rapeseed *in vitro* (Nhut et al., 2003; Li et al., 2013); seedlings of cucumber (Hernández and Kubota, 2016); rosette leaves of rapeseed (Shengxin et al., 2016); fully expanded leaves of lettuce (Wang et al., 2016). It is well known that red light plays important role in shoot elongation and plant anatomy via phytochrome (Schuerger et al., 1997) while blue light is important for Chl biosynthesis (Tripathy and Brown, 1995; Matsuda et al., 2008; Hogewoning et al., 2010; Terfa et al., 2013; Hernández and Kubota, 2016), stomatal development and opening (Hogewoning et al., 2010; Wang et al., 2016). However, very little published information is available on the use of different red- and blue-LEDs combinations to study plants at different growth stages. Furthermore, optimal combination of red- and blue-LED may also depend on the intensity of LED and other light spectra such as far-red and green light. In the study with white clover (*Trifolium repens* L.), Christophe et al. (2006) reported that leaf appearance rate and petiole elongation were strongly controlled by the changes of both blue light and intensity. Fan et al. (2013) used red- and blue-LED to investigate the effects of different light intensities from 50 to 500 μmol m^-2^ s^-1^ on growth and leaf development of young tomato plants. They found that the leaves were thicker, and the stomatal frequency and stomatal area per unit leaf area were higher under PPFD of 300 μmol m^-2^ s^-1^ compared to other light intensities. The highest net photosynthesis rate was also achieved under 300 μmol m^-2^ s^-1^. Their results implied that 300 μmol m^-2^ s^-1^ was more suitable for the culture of young tomato plants. As mentioned earlier, LMA was higher in *M. crystallinum* grown under all combined red- and blue-LEDs than under red-LED alone at PPFD of 350 μmol m^-2^ s^-1^. However, no clear trend in LMA was observed among *M. crystallinum* grown under different LEDs (data not shown). These findings further suggest that effects of combined red- and blue-LEDs on leaf growth of *M. crystallinum* may depend on light intensity. Ooi et al. (2016) reported that the leaf length and area of the first two leaf pairs of *Arabidopsis* grown under a combination of red and blue laser light were higher than the control plants grown under white fluorescent light and equal or lower in the subsequent leaf pairs. Bolting and flowering times of red and blue laser grown plants were slightly delayed, which is most likely due to the absence of far-red light that promotes flowering (Ishiguri and Oda, 1972; Runkle and Heins, 2001). Although far-red as well as green light is photosynthetically inefficient, they exhibit some effects on the growth and development, especially in vegetative development, flowering, stomatal opening, and stem growth modulation (Folta and Maruhnich, 2007; Wang and Wang, 2014). Light spectra and intensities can easily be manipulated by growers. In future studies, intensity of LEDs, other colors of LEDs or laser lights such as far red and green should be supplemented to red and blue lights at different times of the day and different growth stages to investigate their potential application to further enhance plant growth and productivity.

A number of studies have shown that blue light affects photosynthetic performance at the leaf level such as Chl content, Chl *a*/*b* ratio, Chl *a*/*b*-binding protein of PSII, and photosynthetic electron transport (Leong and Anderson, 1984; Senger and Bauer, 1987; López-Juez and Hughes, 1995; Wang et al., 2016). In the study with lettuce grown under mixture of red and blue light or blue light treatments had a higher Chl *a*/*b* compared with red light treatment (Wang et al., 2016). Blue light can be absorbed either by Chl or by Car for the formation of “sun-type” chloroplasts (López-Juez and Hughes, 1995). Compared to those plants grown under 0B condition, all *M. crystallinum* plants grown under different combinations of red- and blue-LED had similar higher levels of total Chl and Car contents (**Figures 4A,C**) and Chl *a*/*b* ratio (**Figure 4B**). These findings suggest that 10% of blue-LED was sufficient to stimulate “sun-type” characteristics (Evans, 1988), even at a rather low irradiance (red:blue LED; 315:35 μmol photon m^-2^ s^-1^). Blue-deficiency that was adverse to Chl biosynthesis has been reported in different plants such as wheat (Tripathy and Brown, 1995), *Rosa* ×*hybrida* (Terfa et al., 2013), spinach (Matsuda et al., 2008), and cucumber seedling (Hogewoning et al., 2010; Hernández and Kubota, 2016). Development of “sun-type” characteristics of *M. crystallinum* plants with blue light enables plants to acclimate to high irradiance (Buschmann et al., 1978; Evans, 1988; Hogewoning et al., 2010). High-light acclimatized leaves normally have higher Chl *a*/*b* ratio with less Chl *b* containing light-harvesting antennae, and thus a higher capacity for electron transport (Leong and Anderson, 1984; Senger and Bauer, 1987; Evans, 1988). In the present study, red- and blue-LED grown *M. crystallinum* plants showed significantly higher qP and ETR than those grown under red-LED alone (0B) from low to high measuring PPFDs (**Figures 5A,C**). qP and ETR measured at a PPFD of 335 μmol photon m^-2^ s^-1^ that was close to the PPFD (350 μmol photon m^-2^ s^-1^) under which all plants were grown, were significantly higher in all red- and blue-LED grown *M. crystallinum* than those grown under 0B condition (**Figures 6A,C**) which is mainly a results of higher photosynthetic utilization of radiant energy since higher levels of total Chl contents and Chl *a*/*b* ratios on a FW basis were found in these red- and blue-LED grown leaves (**Figures 4A,B**). It was noticed that higher blue-LEDs resulted in higher NPQ and ETR measured under higher PPFD (**Figures 5B,C**) or under a PPFD of 335 μmol photon m^-2^ s^-1^ that was similar the growth PPFD (**Figures 6B,C**).

NPQ is a mechanism by which plants protect themselves against excess excitation energy and the resulting photodamage through Car (Demmig-Adams and Adams, 1996; Dreuw et al., 2005). Car serve as a protective mechanism for plants as it absorbs blue light for photoprotection (xanthophyll) and for photoreception in guard cells (zeaxanthin) (Tallman et al., 1997). Hemming (2011) reported that excessive blue light could cause photodamage of PSII and the increase in NPQ could be due to the process of either protecting the leaves from light-induced damage or the damage itself. In this study, combinations of red- and blue-LED, for instance, particularly with higher blue-LED such as 20B, 30B, 50B, and 100B may cause some reversible damage on photosynthetic machinery of *M. crystallinum* and thus resulted in higher Car contents (**Figure 4C**) coupled with higher NPQ (**Figure 6B**). The induction of NPQ depends on a pH gradient (ΔpH) across the thylakoid membranes. Thus, cyclic electron flow around photosystem I (PSI) is another mechanism for dissipating excess photon energy (Heber et al., 1995; Heber, 2002; Makino et al., 2002; Miyake et al., 2004; Yamori et al., 2015).

The light response curves of ETR showed that *M. crystallinum* grown under 100B had much higher values from a PPFD of 155 μmol photon m^-2^ s^-1^ and above compared to those of plants grown under 10B and 0B (**Figure 5C**). When measured at a PPFD of 335 μmol photon m^-2^ s^-1^, ETR values of *M. crystallinum* grown under 30B, 50B, and 100B were higher than those of plants under other lower level of blue-LED indicate that plants under higher blue light treatments utilized more light energy absorbed by Chl for photochemistry (Hemming, 2011; He et al., 2015). Higher ETR of *M. crystallinum* with higher blue-LED treatments (**Figures 5C, 6C**) could be partially due to cyclic electron transport around PSI that is proposed to be essential for protecting both photosystems from the damage (Shikanai, 2007; Miyake, 2010; Takahashi and Badger, 2011). *M. crystallinum* grown under higher blue-LED such as 20B or higher could had spent more energy to protect them from photodamage and/or recover from photodamage and thus did not further increase shoot and root productivity (**Figure 3**). However, all plants were healthy with Chl fluorescence *F*_v_/*F*_m_ ratios of >0.8 (data not shown). These results were similar to our previous studies with Chinese broccoli (*Brassica alboglabra* Baile) under high blue-LED (He et al., 2015). However, Miao et al. (2016) reported that red light alone inhibited electron transport from PSII donor side to PSI whereas decreased *F*_v_/*F*_m_ ratio and PSII were observed in lettuce plants exposed to 100% blue light (Wang et al., 2016). Inhibition of electron transport and deceased *F*_v_/*F*_m_ ratio could result from imbalance of energy allocation between two photosystems (Tennessen et al., 1994).

High-light acclimatized leaves developed under blue light had higher CO_2_-saturated photosynthetic rate measured under higher PPFD and more Calvin cycle enzymes (Leong and Anderson, 1984; Senger and Bauer, 1987; Evans, 1988; Terfa et al., 2013). These are supported by the facts that higher CO_2_-saturated rates of photosynthetic O_2_ evolution (**Figure 7A**), *A*_sat_ (**Figure 7C**), and Rubisco protein (**Figure 8C**) were obtained from *M. crystallinum* grown under different combinations of red- and blue-LED than those of *M. crystallinum* grown under 0B condition. It seemed that *M. crystallinum* grown at 0B exhibited the characteristics of low-light-grown plants with lower CO_2_-saturated rates of photosynthetic O_2_ evolution (**Figure 7A**), less TSP (**Figure 8B**), and Rubisco protein (**Figure 8C**) (Evans, 1988; Terfa et al., 2013). On the other hand, lower CO_2_-saturated rates of photosynthetic O_2_ evolution and *A*_sat_ in *M. crystallinum* grown under 0B could also be due to the loss of photochemical efficiency of PSII as observed in cucumber plants (Hogewoning et al., 2010). There were no significant differences in CO_2_-saturated rates of photosynthetic O_2_ evolution and *A*_sat_ regardless of red- and blue-LED combinations. Conversely, lower photosynthetic activity was observed in cucumber leaves under higher percentage of blue-LED which was postulated to be due to the absence of responses regulated by red light in the stimulation of cryptochrome and phototropin (Hogewoning et al., 2010).

*Mesembryanthemum crystallinum* has been widely studied for its ability to shift from C_3_ photosynthesis to CAM. However, results have showed no differences in CAM acidity across the treatments after grown for 21 days (**Figure 7B**) and all had very low CAM acidity. In the present study, leaf CAM acidity of *M. crystallinum* was about 1.10 μmol H^+^ g^-1^ FW (**Figure 7B**). However, higher accumulations of leaf titratable acidity was recorded in CAM-induced *M. crystallinum* by water stress and abscisic acid treatment, ranging from 20 to 120 μEq g^-1^ FW (Chu et al., 1990). Winter and Holtum (2007, 2014) reported that under carefully managed conditions, well-watered *M. crystallinum* can grow without shifting to CAM. Values of *A*_sat_ and *g*_ssat_ for *M. crystallinum* respectively ranged from 11.4 to 18.3 μmol CO_2_ m^-2^ s^-1^ (**Figure 7C**) and 508.5–667.8 mmol H_2_O m^-2^ s^-1^ (**Figure 7D**) also demonstrated that this facultative CAM plant use C_3_ photosynthesis pathway to fix CO_2_ when there were supplied adequate water under all combinations of red- and blue-LED lightings. Our previous studies showed that C_3_ vegetable crops such as Chinese broccoli (*B. alboglabra* Bailey) and lettuce (*Lactuca sativa*) grown under combinations of red- and blue-LED had similar values of *A*_sat_ and *g*_ssat_ as *M. crystallinum*, ranging from 14 to 20 μmol CO_2_ m^-2^ s^-1^ and 600–800 mmol H_2_O m^-2^ s^-1^, respectively (He et al., 2015, 2016). Plants grown under blue light rich environment had increased *g*_s_ that resulted in greater net photosynthetic rate than those grown under blue light limited conditions (Hogewoning et al., 2010). It was reported that when *M. crystallinum* operated in the C_3_ mode, it responded to blue but not to red light (Mawson and Zaugg, 1994; Tallman et al., 1997). In the present study, similar higher *g*_ssat_ values were obtained from *M. crystallinum* grown under combinations of red- and blue-LED (**Figure 7D**), suggest that 10% of blue-LED (10B) was sufficient for maximal *g*_ssat_ that resulted in maximal *A*_sat_ (**Figure 7C**). Hogewoning et al. (2010) found that the leaves of cucumbers displayed a dysfunctional photosynthetic capacity under 0B, while under 100B the leaves had a low photosynthetic ability. When increasing blue light fraction from 0 to 50%, photosynthetic capacity increased with increasing blue light dose. The similar trends have been reported in rice (Matsuda et al., 2004) and lettuce (Wang et al., 2016). However, in the study with cucumber seedlings, Hernández and Kubota (2016) found that the Chl content, photosynthetic rates, and *g*_s_ increased with the spectra ratio change from 0 to 100%.

The increased Rubisco content under blue-LEDs at high light intensity was correlated with an enhanced photosynthetic rate and faster plant growth than those grown under red-LED (Muneer et al., 2014). High amount of Rubisco can be beneficial for plants since there was a positive relationship of leaf Rubisco contents with light-saturated photosynthetic rates in most C_3_ plants (Makino, 2011; Ashraf and Harris, 2013). The enhancement of Rubisco under high intensity of blue-LED might be associated with an increase in the TRN content (Muneer et al., 2014). The results of the present study clearly showed that leaves of all *M. crystallinum* plants had similar amount of TRN about 65–70 mg g^-1^ DW (i.e., 6–7%, **Figure 8A**); indicating that all plants had more than sufficient amount of N in the leaf tissues. Hogewoning et al. (2010) also reported that N availability did not limit the photosynthetic capacity from 0 to 100% blue lights. However, leaf total soluble and Rubisco proteins of *M. crystallinum* were enhanced under all combinations of red- and blue-LED lighting (**Figures 8B,C**) compared to those plants grown under 0B condition. These findings suggest that *M. crystallinum* grown under blue-LED lighting, had more N allocated to Rubisco protein that could make up more than 50% of Rubisco protein in leaves (Jordon et al., 1992). The enhancement of Rubisco under blue-LED might be associated with an increase in the amount of TRN content and leaf total soluble content (Muneer et al., 2014). However, there was no linear relationship between leaf soluble protein and Rubisco protein and light-saturated photosynthesis. This is because, in the present study, higher percentage of blue-LED further increased Rubisco content (**Figure 8C**) of *M. crystallinum* whereas this was not observed in the total leaf soluble protein (**Figure 8B**) and light-saturated photosynthesis (**Figure 7A,C**). Muneer et al. (2014) suggested that higher Rubisco under blue-LEDs at high light intensity was associated with photosynthetic performance and provided an advantage for higher growth of plants than those grown under red-LED. The role of Rubisco in *M. crystallinum* grown under higher blue-LED lighting merits our further study.

## Author Contributions

JH initiated and funded the expenses for the project, carried out some parts of the experiments and wrote the manuscript. JH, LQ, and TC planned the experiments. LQ and EC carried out most experiments. SL initiated the project.

## Conflict of Interest Statement

The authors declare that the research was conducted in the absence of any commercial or financial relationships that could be construed as a potential conflict of interest.
